# Lab-Scale Cultivation of *Cupriavidus necator* on Explosive Gas Mixtures: Carbon Dioxide Fixation into Polyhydroxybutyrate

**DOI:** 10.3390/bioengineering9050204

**Published:** 2022-05-10

**Authors:** Vera Lambauer, Regina Kratzer

**Affiliations:** 1 Austrian Centre of Industrial Biotechnology (ACIB), Krenngasse 37, A-8010 Graz, Austria; veralambauer@acib.at; 2 Institute of Biotechnology and Biochemical Engineering, Graz University of Technology, NAWI Graz, Petersgasse 12/II, A-8010 Graz, Austria

**Keywords:** non-phototrophic CO_2_ assimilation, *Knallgas* cultivation, *Chemolithotrophs*, ATEX compliant bioreactor, dissolved oxygen control

## Abstract

Aerobic, hydrogen oxidizing bacteria are capable of efficient, non-phototrophic CO_2_ assimilation, using H_2_ as a reducing agent. The presence of explosive gas mixtures requires strict safety measures for bioreactor and process design. Here, we report a simplified, reproducible, and safe cultivation method to produce *Cupriavidus necator* H16 on a gram scale. Conditions for long-term strain maintenance and mineral media composition were optimized. Cultivations on the gaseous substrates H_2_, O_2_, and CO_2_ were accomplished in an explosion-proof bioreactor situated in a strong, grounded fume hood. Cells grew under O_2_ control and H_2_ and CO_2_ excess. The starting gas mixture was H_2_:CO_2_:O_2_ in a ratio of 85:10:2 (partial pressure of O_2_ 0.02 atm). Dissolved oxygen was measured online and was kept below 1.6 mg/L by a stepwise increase of the O_2_ supply. Use of gas compositions within the explosion limits of oxyhydrogen facilitated production of 13.1 ± 0.4 g/L total biomass (gram cell dry mass) with a content of 79 ± 2% poly-(*R*)-3-hydroxybutyrate in a simple cultivation set-up with dissolved oxygen as the single controlled parameter. Approximately 98% of the obtained PHB was formed from CO_2_.

## 1. Introduction

CO_2_ emissions from fossil fuel combustion and further anthropogenic activities are the largest driver of global warming. Stripping the CO_2_ back out of waste streams and using it as carbon feedstock would close the carbon loop and spare fossil fuels. Therefore, CO_2_ emissions would be limited and climate change impacts mitigated. One major obstacle in CO_2_ reuse is the high stability of the molecule. Considerable energy input is needed to activate CO_2_ for further use and large-volume CO_2_ conversion processes are currently limited to a few industrial processes (e.g., urea, methanol, carbonate, and formic acid production) [1,2]. Nature has evolved highly sophisticated mechanisms for carbon fixation and utilization. The reducing energy for CO_2_ reduction is either obtained from light-dependent reactions or from oxidation of inorganic compounds. Aerobic hydrogen-oxidizing bacteria (HOBs or *Knallgas* bacteria) assimilate CO_2_ by H_2_ oxidation. Provision of the reducing power by H_2_ oxidation is an efficient route in non-phototrophic CO_2_ assimilation and promises higher growth rates while promoting drastically less land, fresh water, and mineral requirements as compared to photosynthetic organisms [3,4]. The HOB *Cupriavidus necator* was compared to the green microalga *Neochloris oleoabundans* in microbial CO_2_ fixation. *C. necator* has been reported to exhibit 3–6 times higher energy efficiencies and higher biomass yields in comparison to *N. oleoabundans* in microbial CO_2_ fixation [5]. *C. necator* can accumulate polyhadroxyalkanoate as carbon storage to levels of up to 82% of the cell’s dry weight (on CO_2_ under chemolithotrophic conditions) [6,7]. The metabolism is tractable by genetic engineering, and alternative products from CO_2_ such as tailor-made polyhydroxyalkanoates or versatile organic solvents become available [8,9,10,11]. However, applications of *C. necator* and other HOBs for CO_2_ fixation are still hesitant despite the increasing demand for cheap feedstocks. The largest published HOB cultivation of 23 L was reported in 1976 [12]. The main reason for slow implementation of chemolithotrophic cultivations is technical hurdles, and thus high implementation costs due to the explosiveness of H_2_ and O_2_ mixtures (low ignition energy ≥ 0.016 mJ). Gases must be mixed on-site (requiring several gas lines), labs need safety measures (gas sensors, check-valves, ventilated hoods, explosion doors, antistatic workwear, etc.), and equipment must be explosion-proof (avoidance of ignition sparks and electrostatic discharges). Construction measures are time-consuming and explosion-proof equipment is, as a rule of thumb, at least 10 times more expensive than standard equipment (magnetic stirrers, bioreactors etc.). Finally, some residual uncertainty regarding lab safety remains despite all safety measures. In the last several years, a restricted number of labs equipped for fermentations of explosive gas mixtures were reported (with no claim to completeness [10,13,14,15,16,17]). In parallel, several groups engineered HOB strains (first and foremost *C. necator*). However, labs focusing on molecular biotechnology are generally not equipped for oxyhydrogen cultivations (exceptions e.g., [10,11,13,18]). Therefore, newly developed strains were often grown in closed jars or bottles flushed with gases once or twice a day (e.g., [8,9,16,19]). Obviously, fast-growing HOB strains are nutrient-limited under these conditions and strain characterization is affected (difficulties in measuring growth and nutrient levels).

In the present study, we report on reproducible, safe, and easy-to-perform chemolithotrophic cultivations of HOBs. Conditions for long-term strain maintenance of *C. necator* H16 were optimized and mineral media composition for chemolithotrophic cultivation was studied. An ATEX compliant cultivation system was developed and used to cultivate *C. necator* on a gram scale. We note that ATEX directives are EU directives describing the minimum safety requirements for workplaces and equipment used in explosive atmospheres. Dissolved oxygen concentration (DO) is generally considered the most critical parameter in the autotrophic cultivation of HOBs [6,20]. Here, we compared different oxygen supply strategies: a constant oxygen supply adjusted to the oxygen need of the inoculum, a stepwise increased oxygen supply guided by the biomass formation over time, and a finely tuned oxygen supply guided by an implemented O_2_ dipping probe measuring the DO concentration. Our results pave the way towards a broader use of efficient, microbial CO_2_ assimilation.

## 2. Materials and Methods

### 2.1. Chemicals, Enzymatic Assays and Strains

The tryptic soy broth (TSB, CASO Buillon, X938.2) was from Carl Roth (Karlsruhe, Germany). The kanamycin sulfate (≥750 I.U./mg, T832.1), ampicillin sodium salt (≥97%, K029.5), chloramphenicol (≥98.5%, 3886.1), geneticin disulfate (Bio-Science grade, 2039.2), and erythromycin (≥98.0%, 4166.1) were from Carl Roth. The tetracycline (≥98.0%, 87128) was from Fluka (Vienna, Austria) and poly-(*R*)-3-hydroxybuttersäure (quality level 200, 363502) from Sigma-Aldrich (Vienna, Austria). Other chemicals were from Sigma-Aldrich/Fluka or Carl Roth and were of the highest purity available. An enzymatic assay for ammonium (K-AMIAR) was obtained from Megazyme International (Wicklow, Ireland). The strain *C. necator* H16 DSM 428 (aka ATCC 17699, NCIB 10442) was from DSMZ, Deutsche Sammlung für Mikroorganismen und Zellkulturen.

### 2.2. Growth Media

The TSB media was prepared with 30 g/L TSB supplemented and 50 mg/L kanamycin sulfate, if not mentioned otherwise (note that no additional C-source was added to TSB media). The mineral media (MM) was prepared as described by Atlić et al. [21] (Table 1) with small modifications. Kanamycin sulfate (end concentration 50 mg/L) was added to part 1 prior to autoclaving. Parts 1 to 5 were autoclaved separately, and part 6 was sterile filtered (0.2 μm). Parts were combined prior to cultivation. For media with B12, 0.01 to 10 mg/L B12 (sterile filtered) was added to the media. In chemolithotrophic cultivations, 2 g/L fructose were added. For agar plates, mineral media with 20 g/L agar-agar and 10 g/L fructose was used.

### 2.3. Heterotrophic Cultivations

For precultures, 300 mL baffled flasks (50 mL TSB or MM with fructose) were inoculated from agar plates and incubated at 30 °C and 110 rpm (Rotary shaker CERTOMAT BS-1) for 24 h. Main cultures (300 mL baffled flasks, 50 mL TSB media, or MM with fructose) were inoculated with 1 mL of preculture to start optical densities 600 nm (OD_600_) of ~0.2 and incubated at 30 °C and 110 rpm for 2 days, unless otherwise stated.

#### 2.3.1. Antibiotic Resistances

TSB main cultures (300 mL baffled flasks) were used to study antibiotic resistances. The media was supplemented with six antibiotics, each applied at three different concentrations: kanamycin (50; 25; 10 mg/L), ampicillin (115; 58; 23 mg/L), chloramphenicol (34; 17; 6.8 mg/L), geneticin (50; 25; 10 mg/L), tetracycline (50; 25; 10 mg/L), and erythromycin (30; 15; 6 mg/L). All antibiotics were sterile filtered (0.2 μm) and added to the autoclaved media. Cultures without antibiotics served as reference. The OD_600_ was measured after 48 h. Experiments were done in duplicates.

#### 2.3.2. Comparison of TSB and MM

Precultures and main cultures were prepared with the respective media in 300 mL baffled flasks (50 mL TSB or MM with fructose). Experiments were done in duplicates.

#### 2.3.3. Effect of Cultivation Temperature on Growth

Precultures were cultivated in 300 mL baffled flasks with 50 mL MM and incubated at 30 °C and 110 rpm for 24 h. Main cultures were either cultivated in 1 L baffled shaken flasks (with a magnetic bar of 70 × 10 mm, 250 mL of MM) or in a 1 L DURAN^®^ GLS 80 wide-neck threaded glass bottle with a magnetic anchor stirrer (from DWK Life Sciences purchased at Roth, Germany) (950 mL MM). Mixing was accomplished by stirring at 400 rpm on magnetic stirrers. Main cultures were inoculated to OD_600_ of ~0.2. Cultures in 1 L baffled flasks were stirred at room temperature (22–24 °C) and 30 °C. The DURAN^®^ GLS 80 bottle was stirred at room temperature. The OD_600_ was measured over time. Experiments were done in duplicates.

#### 2.3.4. Determination of Optical Density (OD_600_) and Cell Dry Mass (CDM)

The wavelength for optical cell density measurements was set to 600 nm according to wavelength scans of pure TSB and MM media (Figure S1 in the Supplementary Information). Cell dry mass (CDM) was determined gravimetrically. Note that there was no differentiation between microbial biomass and stored polyhydroxybutyrate. The correlation factor of g_CDM_/OD_600_ was 0.246 with a R² of 0.965 (Figure S2 in the Supplementary Information).

### 2.4. Preservation of C. necator H16

For short-term storage of up to 1 month, cells were maintained on TSB or minimal medium agar plates at 4 °C. For long-term storage, cryoservation solutions containing glycerol, trehalose, and DMSO were tested. Stock solutions of glycerol, trehalose, and DMSO were mixed with the cell suspension (overnight cultures in TSB media) to end concentrations of 17%, 25%, and 33% glycerol, 0.8 M trehalose, 17% glycerol + 0.6 M trehalose, or 50% DMSO. Cryoservation stocks (2 mL free-standing cryogenic vials, with outer thread from Carl Roth) were frozen in liquid N_2_ and stored at −80 °C. Every second month, 300 mL baffled flasks with 50 mL TSB media were inoculated with 75 μL cells from cryoservation stocks. After 24 h of cultivation, the OD_600_ was measured.

### 2.5. Chemolithotrophic Cultivation (Oxyhydrogen Cultivation)

**Figure 1:**
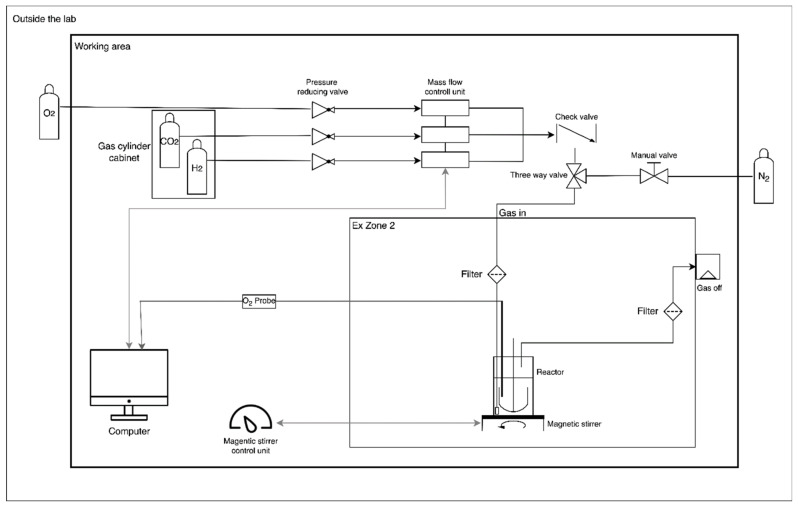
Scheme of gas cultivation setup. Flow diagram of installations and cultivation equipment. (Open-source program diagrams.net © 2005–2021 JGraph Ltd. was used for figure preparation).

Explosion safety to ensure personal and technical safety was considered according to Austrian and European standards (ATEX directive RL 1999/92/EG and RL 2014/34/EU). In Figure 1, a detailed scheme of the ex-safe cultivation set-up is displayed. Gas lines for the mixed substrate gas, the interior of the bioreactor, and the off-gas line were defined as ex-zone 0 (the area in which an explosive atmosphere is present continuously or for long periods). The remaining interior of the room and fume hood was defined as ex-zone 2 (no ex-zone 1 was defined due to strong ventilation by the fume hood, see below).

#### 2.5.1. Installations and Equipment

The room had an antistatic floor and a pressure relief flap (40 × 40 cm). The room was equipped with two gas cylinder cabinets with pressure-reducing valves (O_2_ and H_2_ separated, cylinder cabinet for H_2_ ventilated with 60 m³/h), gas lines and gas mass flow controllers for CO_2_, H_2_, O_2_ (MFCs; red-y smart controller from Vögtling Instruments GmbH, Switzerland), and an additional gas line for the inert gas N_2_. Gas flow rates of CO_2_, H_2_, and O_2_ were adjusted online by the respective MFCs using the get red-y software (from BURDE•CO GmbH, Vienna, Austria). A RF 53 series check valve (Wittgas Gastechnik GmbH & Co KG, Witten, Germany) was installed directly after the gas mixer. The N_2_ was connected to the substrate gas line by a three-way valve to enable purging of the bioreactor prior to sampling. All gas cultivations were done under explosion-safe conditions in an ex-safe fume hood (559 m³/h; Secuflow from Waldner, Germany, Wangen, for use in zone 1 II2G/Gb according to ATEX directive RL 1999/92/EG). The fume hood had an H_2_ sensor coupled to an automatic 2/2-way magnetic valve (Tescom Europe Selnsdorf, Germany). Experimenters wore safety glasses, antistatic lab coats, antistatic shoes, and a portable gas detector (MSA Multi-Gaswarngerät Altair 4XR, Schloffer Arbeitsschutz GmbH, Hart bei Graz, Austria).

**Figure 2:**
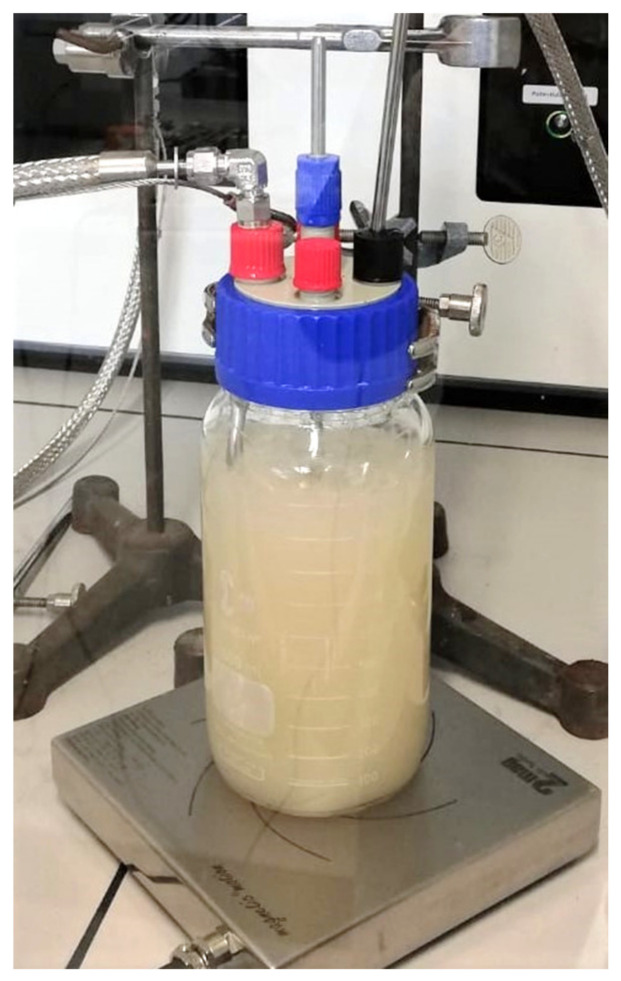
Explosion-proof bioreactor setup consisting of a 1 L DURAN^®^ bottle with a magnetic anchor stirrer on a magnetic stirrer plate. Gas substrate is supplied by a steel tube with a PTFE frit at the end.

The gas cultivation setup is displayed in Figure 2. The substrate gas passed H_2_-tight, autoclavable, polyvinylidene fluoride filters (filter #12.32.5K 99.99% removal of 0.1 micron particles, stainless steel housing #SS117.201 purchased at BURDE•CO) prior and subsequent to the cultivation vessel. Off-gas was drained into the fume hood exhaust air. As the cultivation vessel, a stirred 1000 mL DURAN^®^ GLS 80 wide-neck threaded glass bottle was used. A stirrer reactor cap GLS 80 with a magnetic anchor stirrer was attached (from DWK Life Sciences purchased at Roth, Germany). A magnetic stirrer atexMIXdrive (2 mag AG, Munich, Germany) ensured explosion-proof stirring. The substrate gas mixture was supplied through a 6 mm steel tube connected to a 5 mm silicon tube ending in a PTFE frit (product code 01018-22707, Agilent Technologies Österreich GmbH, Vienna, Austria). Online monitoring of dissolved oxygen (DO) was established using an oxygen-dipping probe DP-PSt3 from PreSens GmbH, Regensburg, Germany. The oxygen-dipping probe consisted of a polymer optical fiber (coated with an oxygen-sensitive foil at the end), covered in a high-grade steel tube (35 cm) to facilitate fitting of the probe into the reactor. The steel tube was connected to an equipotential socket in the hood. Read-out electronics were outside of the ventilated hood (facilitated by the 2.8 m fiber-optical cable). No ignition sources were in the ventilated hood. The outlet for the off-gas was a steel pipe connected to a gas-tight, flexible tube.

#### 2.5.2. Determination of the Volumetric Oxygen Transfer Coefficient (*k*_L_*a*)

The *k*_L_*a* of the bioreactor was determined with the “static gassing-out” method [22]. The DO concentration was measured with the O_2_-dipping probe in MM without cells (see also Figures S3 and S4 in the Supplementary Information). First, the media was gassed with O_2_ for 30 min. The subsequently measured DO value represented the maximum amount of O_2_, referred to as O* and 100% oxygen saturation. (The read-out of the dipping probe was % oxygen saturation.) Next, the media was degassed with N_2_ for 30 min. Then, the O_2_ gas flow was set to 100, 200, and 400 mL/min at 340 rpm stirrer speed. The increasing DO concentration was measured over time and recorded (as % oxygen saturation).

The *k*_L_*a* was determined using the integrated form of Equation (1).

(1) $$\frac{\mathrm{dDO}}{\mathrm{dt}}=k_{L}a\left(O^{*}-\mathrm{DO}\right)\;\mathrm{integration}\;\to \;\ln\left(O^{*}-\mathrm{DO}\right)=-k_{L}a\;t$$

The response time of the O_2_-dipping probe (t_90_) was <40 s as specified by the manufacturer.

#### 2.5.3. Calculation of Henry’s Law Constant H^cp^ and Oxygen Transfer Theory

O* is proportional to the partial pressure p_O__2_ of O_2_ in the gas mixture and the Henry’s law constant H^cp^ (1.3 · 10^−3^ M/atm at 25 °C in pure water) as a proportionality factor (Equation (2)).

(2) $$O^{*}={\;H}^{\mathrm{cp}}p_{O2}\;$$

In a typical cultivation medium, O_2_ solubility is considered 5 to 25% lower than in water. Here, we calculated a H^cp^ of 1.18 mM/atm (according to Schumpe et al. [23]).

The actual concentration of the DO in the bioreactor is a dynamic value constituted by the oxygen transfer rate (OTR) and the oxygen uptake rate (OUR). The OTR depends on O* (equilibrium O_2_ concentration), the liquid phase mass transfer coefficient *k*_L_ and the specific exchange area *a* (gas–liquid interfacial area per unit of fluid) (Equation (1)).

The OUR depends on the biomass concentration X and the specific O_2_ consumption rate q_O2_ (Equation (3)).

(3) $$\frac{\mathrm{dn}}{\mathrm{dt}}={\;q}_{O2}X\;=\;\mathrm{OUR}$$

In a quasi-steady state, the OTR equals the OUR (Equation (4)).

(4) OTR = OUR

#### 2.5.4. Gas Cultivations

Gas cultivations were done either with constant gas flow rates (low and high p_O2_), with manually increased oxygen supply guided by the biomass concentration (biomass concentrations measured from samples taken over time) or with manually increased oxygen supply guided by the DO concentration (in situ measurement of DO concentration using the O_2_-dipping probe, referred to cultivations 1 to 4 in the text).

For all chemolithotrophic experiments, precultures were cultivated heterotrophically in MM with fructose overnight (end OD_600_ ~ 9). For the main culture, the autoclaved 1 L DURAN^®^ bottle was filled with 950 mL MM and 50 mL preculture under sterile conditions (start OD_600_ < 1.4). If used, the O_2_-dipping probe was chemically sterilized with ethanol 70% (*v*/*v*) and fit into the reactor. The filled bioreactor was connected to the substrate gas and off-gas lines. Tightness of connections was checked with the purge gas N_2_ and a leak detector spray. H_2_, CO_2_, and O_2_ gas flows (NmL/min) were adjusted online. Total flow rates of 100 to 400 NmL/min were used. Two to three samples were taken per day. Prior (and subsequent) to opening the cultivation vessel, the substrate gas was switched off and the whole system was purged with N_2_ for 5 min. Samples were taken with a syringe with a 120 mm needle. Gas supply was summarized in Table 2. Note that individual partial pressures (and hence dissolved gas concentrations) do not depend on total gas flows according to Dalton’s law.

In experiments with increased oxygen supply, the O_2_ flow rate was manually adjusted using the get red-y software of gas mass flow controllers.

#### 2.5.5. Sample Analysis with PHB Quantification

All samples were taken in duplicates. OD_600_ of samples was measured in quadruplicates. 5 mL of cell culture was transferred into 15 mL Sarstedt tubes and centrifuged for 30 min. The supernatants and pellets were stored at −20 °C for further analysis. The ammonium concentrations were determined in the supernatant with the Megazyme assay kit K-AMIAR according to the supplier’s manual. PHB content was analysed on GC. Samples were prepared according to Atlić et al. [21] with small modifications. Pellets were lyophilized and suspended in at least 10-fold pure EtOH (96%) (for small pellets, more EtOH was used to achieve sufficient mixing). Samples were stirred on a magnetic stirrer for 1 day at room temperature. After centrifugation for 30 min at 5000 rpm, the supernatant was discarded. Samples were dried at 70 °C for 2 h, transferred into glass vials with 30-fold chloroform (*w*/*w*) and stirred for 1 day at room temperature. PHB was precipitated by the addition of ice cold EtOH (96%) and filtered through a Whatman filter paper (HK26.1, Carl Roth). The filter cake was dried overnight at room temperature under the fume hood. Dry PHB was scratched from the weighted filter paper and transferred into new glass tubes. At this step, 11 ± 2% of purified PHB was lost. 2 mL of esterification mixture (94.9 mL MeOH, 5 mL H_2_SO_4_, 0.108 mL hexanoic acid, and 2 mL of chloroform were added, and the glass tubes were tightly closed. For transesterification, the samples were shaken at 95 °C in a water bath for 3.5 h. Tubes were cooled down to room temperature and 1 mL of NaHCO_3_ (10% *w*/*v*) was added. Samples were vortexed for 5 min. After 15 to 30 min, the organic phase was transferred into a GC vial using a glass Pasteur pipette and closed tightly. PHB content was analysed on GC using a ZB-5 column (Phenomenex, 30 m length, 0.32 mm inner diameter and 0.25 μm film thickness) and an FID detector according to Juengert et al. [24]. As reference, a standard curve with pure PHB was prepared (R² 0.9999). Standards were treated like the isolated PHB. PHB content in percent per CDM was calculated. The 11 ± 2% weight loss of PHB during transfer from the filter paper into the glass vial was considered. During the transfer from the Sarsted tube into the glass vial, less than 1% of cell dry matter was lost and not considered for PHB calculations.

## 3. Results

### 3.1. Heterotrophic cultivation

#### 3.1.1. Antibiotic Resistances of *C. necator* H16

**Figure 3:**
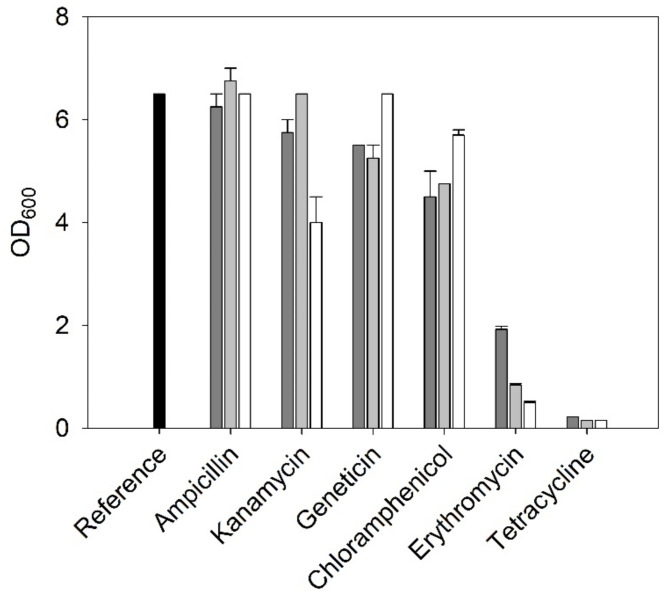
OD_600_ values of *C. necator* cultures in the presence of six antibiotics. Dark gray bars illustrate lowest, light gray bars medium, and white bars highest antibiotics concentrations. Black bar refers to reference cultures without antibiotics. (2 days of cultivation in TSB at 30°C.).

Addition of antibiotics is the most common strategy in the cultivation of microorganisms to prevent growth of contaminating microbes. Here, we decided to add an antibiotic as the bioreactor had to be opened for sampling. A range of strain-dependent resistances to antibiotics have been reported for native *C. necator* strains [25,26,27]. Fast adaptivity of *C. necator* might also lead to altered antibiotic resistances [28,29]. Therefore, resistances of the used *C. necator* H16 strain against six antibiotics generally used in the cultivation of microorganisms were probed, and the effect on culture density quantified after 2 days of cultivation (Figure 3). The addition of 115, 57.5, or 23 mg/L ampicillin and 50, 25, or 10 mg/L kanamycin or geneticin did not significantly affect growth. The presence of 34, 17, or 6.8 mg/L chloramphenicol slightly reduced OD_600_ after 2 days. OD_600_ of ~70% compared to the reference culture was obtained at the highest chloramphenicol concentration. The addition of 30, 15, or 6 mg/L erythromycin diminished culture densities to between 8 and 32% as compared to the reference. The presence of 50, 25, or 10 mg/L tetracycline totally inhibited growth. Kanamycin was chosen as the most suitable antibiotic in all further experiments for the following reasons: it does not inhibit the growth of *C. necator* at a concentration of 50 mg/L, it can be added to the medium prior to autoclaving, and it is used as a standard antibiotic in biotechnological labs.

#### 3.1.2. Heterotrophic Cultivations Using Mineral Media (MM)

**Figure 4:**
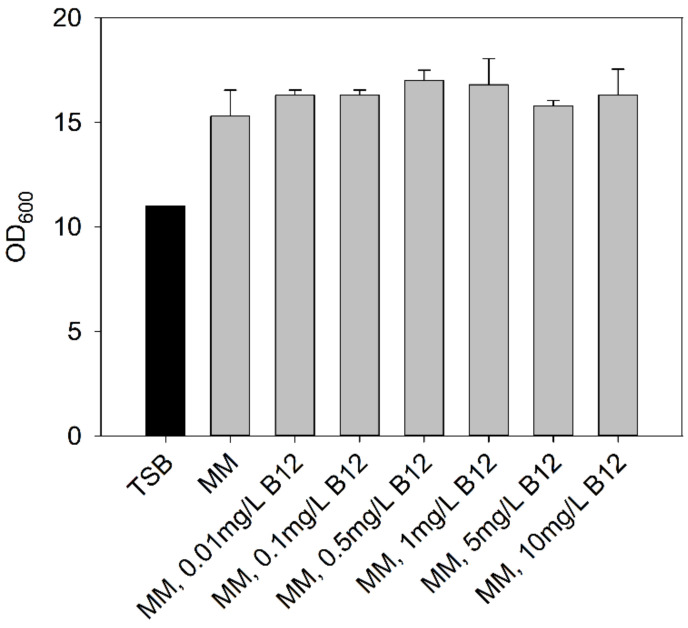
OD_600_ values of *C. necator* cultures in MM supplemented with B12. Black bar refers to cultures in TSB. (2 days of cultivation at 30 °C.)

Chemolithotrophic growth facilitates formation of biomass from CO_2_, H_2_, O_2_, and ammonium salts without a further carbon source. Here, we used a mineral medium reported for heterotrophic cultivations of *C. necator* described by Atlić et al. [21] supplemented with tungsten (recommended by Cramm, [30]) (Table 1). As a C-source, 20 g/L fructose was added to the mineral medium. After 2 days of cultivation, OD_600_ values reached 19 and 11 with MM and TSB media, respectively. Motivated by a notion in Pohlmann et al. [31] that *C. necator* H16 is not able to produce vitamin B_12_ (though it can bypass vitamin B_12_-dependent reactions), we added 0.01 to 10 mg/L of vitamin B12 to the MM with fructose. No effect of B12 supplementation was seen (Figure 4).

#### 3.1.3. Effect of Temperature on Growth Rate

Temperature control in an explosive atmosphere adds complexity to the cultivation system. Autotrophic cultivation at room temperature without temperature control was hence aimed at. Comparison of microbial growth in heterotrophic cultivations performed at room temperature (22–24 °C) and at 30 °C was used to study the effect of lower temperature on microbial growth. The maximum growth rate in MM was 1.4 times higher at 30 °C as compared to room temperature (μ_max,30°C_ 0.17 ± 0.01 versus μ_max,RT_ 0.13 ± 0.01 h^−1^). After 3 days, all cultures had reached optical densities (OD_600_) of 37 (for growth curves and rates, see Supplementary Information Table S1 and Figure S5).

#### 3.1.4. Strain Maintenance

**Figure 5:**
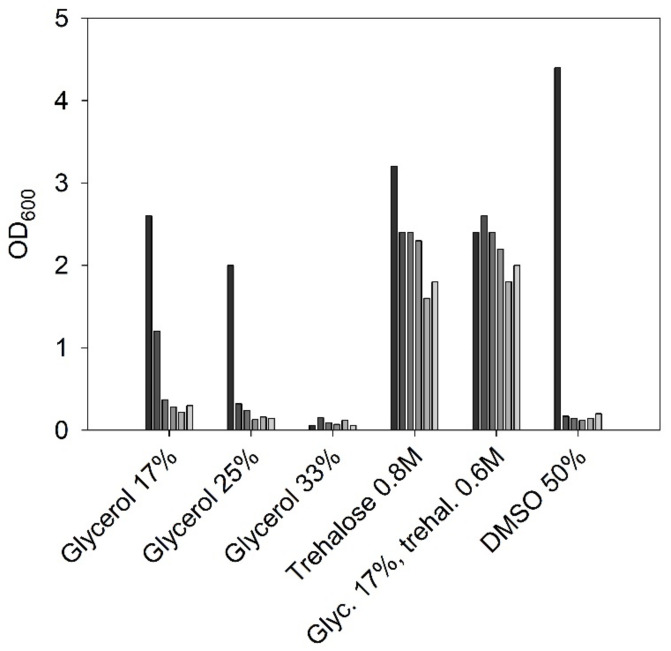
OD_600_ values of *C. necator* cultures inoculated directly with cells from cryoservation stocks. Bars indicate recultivability after 1, 3, 5, 7, 9, 11 months (starting with black bars, descending grey intensities). (1 day of cultivation in TSB at 30 °C).

Setup of a reproducible oxyhydrogen cultivation system for *C. necator* requires stable short-term and long-term strain maintenance. Cultures grown on agar plates were storable at 4 °C for at least 4 weeks. Low recultivability from cryostocks kept at −80 °C with glycerol as cryoprotectant was observed. Therefore, recultivability of cells from cryostocks containing different cryoprotectants was tested. Recultivability after 1 to 11 months of cells stored in glycerol (33%, 25%, 17%), trehalose (0.8 M), DMSO (50% *v*/*v*), and glycerol and trehalose (17% glycerol, 0.6 M trehalose) is displayed in Figure 5. After 11 months, the highest recultivability in 0.8 M trehalose and 0.6 M trehalose with 17% glycerol was experienced.

### 3.2. Chemolithotrophic Cultivation

*C. necator*, as a HOB, forms biomass from CO_2_ as a carbon source using H_2_ as an electron donor and O_2_ as an electron acceptor. Reported substrate gas mixtures indicate ratios of 7:1:1, 7:2:1, or 65:25:10 (reviewed in 10). The two most evident problems connected with substrate gas mixtures containing H_2_ and O_2_ are their high explosion capability and low water solubility. Gases must be dissolved in the aqueous medium to be available for the cells, and the parameters of interest are therefore the dissolved gas concentrations [32]. The O_2_ concentration is of particular importance as growth of *C. necator* is reported to be inhibited at DO > 11.5 mg/L [33]. Previous results have indicated an extended lag phase when the DO was > 3 mg/L. The highest values for μ (and q_O2_) were observed with DO concentrations of ~2.6 mg/L [20].

The actual concentration of DO in the bioreactor is a dynamic value constituted by the oxygen transfer rate (OTR) and the oxygen uptake rate (OUR). An increasing biomass concentration at constant O_2_ supply leads to a decrease in the dissolved O_2_ concentration over time. For optimal growth, the O_2_ supply (OTR) needs to be increased to compensate for the increased biomass concentration and increased OUR.

#### 3.2.1. Chemolithotrophic Cultivations with Constant Gas Flow

**Figure 6:**
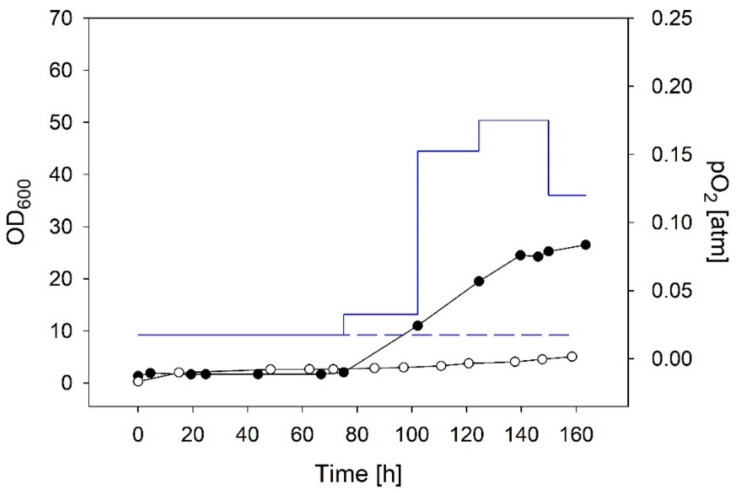
Time curves of gas cultivations with constant gas flow and stepwise increased p_O2_. Circles indicate OD_600_ values, blue lines p_O2_ (constant gas flow white circles and dashed line; stepwise increased p_O2_ black circles and full line).

The substrate gas mixture was composed of H_2_:CO_2_:O_2_ in a ratio of 90:8:2 (total flow rate 400 NmL/min) and kept constant over the entire cultivation time. Gas supply was switched on after transfer of media (MM with 2 g/L fructose) and heterotrophically grown inoculum into the stirred bioreactor. A μ_max_ of 0.008 h^−1^ and a final OD_600_ of 5 (1.2 g_CDM_/L) were obtained after 158 h (Figure 6) (μ_max_ determination Supplementary Information Figure S6). The applied gas composition resulted in a p_O2_ of 0.0175 atm. According to Equation (2), with a H^cp^ of 1.18 mM/atm, the O* was calculated to be 0.7 mg/L. The linear shape of the growth curve indicated O_2_ limitation. However, gas cultivations starting with a relatively high O* of ~1.6 mg/L did not lead to chemolithotrophic growth of *C. necator* after 50 h (Supplementary Information Figure S7).

#### 3.2.2. Chemolithotrophic Cultivations with Stepwise Increased Gas Flow Guided by the Biomass Concentration

Samples were taken over time, OD_600_ values were measured, and the biomass concentrations were calculated. An average q_O2_ of 1.4 mmol g_CDM_^−1^h^−1^ was anticipated because this value was at the lower end of q_O2_ values reported by Lu and Yu (2019) [15] and ensured low DO concentrations (below toxic levels) in autotrophic cultivations. OURs were calculated by multiplying current biomass concentrations (obtained from samples) with the average q_O2_ (Equation (3)). Assuming a quasi-steady state, the OTR equalled OUR (Equation (4)). The DO was set to zero which simplifies OTR to the product of *k*_L_*a* and O* (Equation (1)). The required O* was adjusted by the partial pressure of O_2_ mass flow (Equation (2); note that the O_2_ gas flow was reduced at 150 h as biomass formation rate decreased.) After 163 h, a final biomass concentration of 6.5 g_CDM_/L (OD_600_ 26.5) and a μ_max_ of 0.055 h^−1^ were obtained (Figure 6, μ_max_ determination Supplementary Information Figure S6).

#### 3.2.3. O_2_ Supply Guided by an O_2_ Sensor

**Figure 7:**
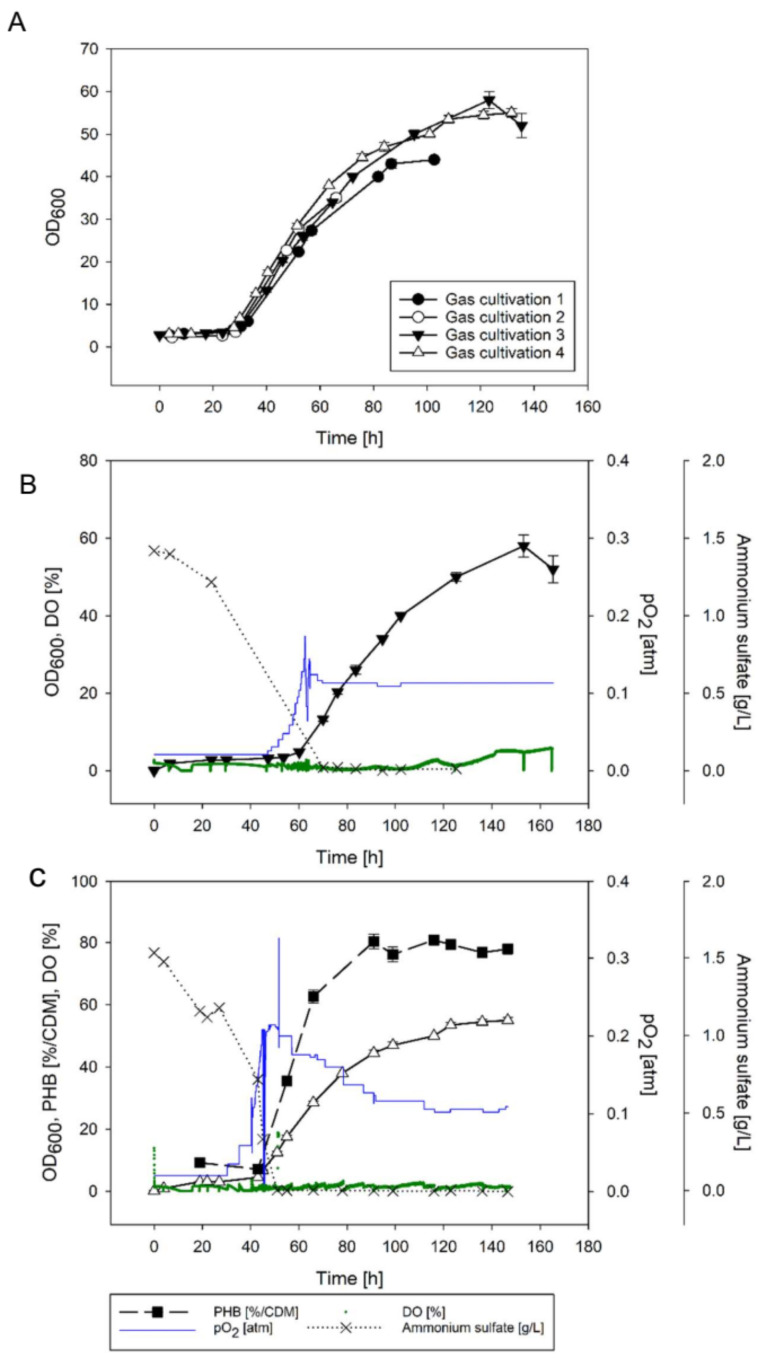
Time curves of gas cultivations with manual DO control using a dipping probe. Panel (**A**) indicates OD_600_ values over time of all four gas cultivations (starting points of gas cultivations in panel A were normalized to the start of CO_2_ assimilation). Panels (**B**,**C**) indicate gas cultivations 3 and 4, respectively (triangles indicate OD_600_ values from Figure 7A, p_O2_ blue line, DO in % (100% corresponds to full saturation with pure oxygen) illustrated as green dots, ammonium black crosses, PHB in % per CDM black squares). All data of gas cultivations are summarized in the Supplementary Data and the Supporting Information Figure S8.

Four gas cultivations with manually stepwise increased O_2_ mass flows guided by the DO concentration were performed (Figure 7A; all data of gas cultivations are summarized in the Supplementary Data). The starting substrate gas mixture in all four cultivations was composed of H_2_:CO_2_:O_2_ in a ratio of 85:10:2 (total flow rate 97 NmL/min, p_O2_ of 0.02 atm). The O_2_ consumption (seen as a drop in DO concentration) gave information about the consumption of C sources. The fructose was used up in the first ~20 h (e.g., in cultivation 4, the fructose was used up after 19 h at a cell dry mass of 0.76 g_CDM_/L; data not displayed). During a lag phase in O_2_ consumption of ~24 h, the cellular metabolism switched from heterotrophy to lithotrophy. CO_2_ assimilation was marked by a drop in the DO. From then, the O_2_ supply was manually increased over time but kept below ~1.1 mg/L. The p_O2_ was 0.12 to 0.15 atm after 50 to 60 h in all four cultivations at constant H_2_ and CO_2_ flow rates. After ~60 h of cultivation, biomass concentrations were ~2 g_CDM_/L and the ammonium was depleted (Figure 7B,C, Supplementary Information Figure S8). Growth curves indicated remarkable reproducibility and a mean maximal growth rate of 0.088 ± 0.010 h^−1^ (Figure 7A; determination of μ_max_ Supplementary Information Figure S6). After 60 to 70 h of fermentation, growth rates decelerated. In cultivation 1, the H_2_ and CO_2_ flow rates were kept constant (Figure 7A and Supplementary Information Figure S8). In cultivations 2 (Supplementary Information Figure S8), 3 (Figure 7B), and 4 (Figure 7C), H_2_ and CO_2_ were increased to 200 and 20 NmL/min after 60 h to avoid H_2_ limitations. In all cultivations, the O_2_ flow rates were further increased after 60 h, but due to increasing DO concentrations, the O_2_ flow rates were back regulated again. Increases of H_2_ and CO_2_ gas flow rates to 200 and 20 NmL/min, respectively, had no significant effect on final cell dry mass (cultivations 3 and 4 in Figure 7B,C, cultivation 2 in Supplementary Information Figure S8). Final OD_600_ values of 53.5 ± 1.5 were obtained after ~150 h. The OD_600_ correlated to a mean cell dry mass of 13.1 ± 0.4 g_CDM_/L (mean value and deviation from the mean for gas cultivations 3 and 4). The PHB content in biomass samples was determined for cultivation 4. The PHB content started at 9% and steadily increased after the ammonium was depleted to 80% after 90 h (Figure 7C). The PHB content was constant (±1.6%) until the end of the gas cultivation.

#### 3.2.4. pH Development in Chemolithotrophic Cultivations

In classic bioreactor systems, pH is monitored and computer-controlled by the addition of acid and base. pH-stat systems are equipped with a pH-probe and two peristaltic pumps for the correcting liquids. A value of 6.8 was previously used in heterotrophic and chemolithotrophic cultivations of *C. necator* (e.g., [21,34]). In the present chemolithotrophic cultivations, no correction of pH was required. The used MM media with fructose had a starting pH value of 6.8. pH values between 6.8 and 7.0 were measured in all samples taken from chemolithotrophic cultures.

## 4. Discussion

### 4.1. Chemolithotrophic Cultivation

Gas compositions vary widely in reported gas cultivations [12,15,35,36,37,38]. The decisive parameters are, however, the concentrations of gases that are dissolved in the aqueous phase and available for the cells [32]. CO_2_ dissolves easily in water (~1.4 g/L) but H_2_ and O_2_ have low solubilities of 1.6 and 40 mg/L, respectively (data for pure water at 25 °C and p_i_ of 1). In the presented experiments, we used excesses of H_2_ and CO_2_ under O_2_-limiting conditions. The DO concentration is one of the most crucial parameters in the cultivation of *C. necator* [6,20,33,34]. The window of operation for DO concentrations in gas cultivations is relatively small. Due to the rapid growth of HOBs, their oxygen consumption increases rapidly over time. A constant oxygen supply adjusted to the oxygen need of the inoculum lead to fast oxygen limitation and a final OD_600_ of 5 (Figure 6). A stepwise increase guided by the biomass concentration (sampling offline) resulted in a final OD_600_ of 26.5, and a stepwise increase guided by the in situ measured DO resulted in a doubled OD_600_ of 56.5. Our findings are briefly summarized in Table 3.

#### 4.1.1. Biological Limitations of DO Concentration

The organism *C. necator* displays an upper limit of 30 kPa (DO < ~11.5 mg/L) for O_2_ when grown under chemolithotrophic conditions [33]. Previous results have indicated an extended lag phase when the DO was >3 mg/L. The highest values for μ (and q_O2_) were observed with DO concentrations of ~2.6 mg/L. At DO concentrations of <1.9 and ≥7 mg/L, significantly lower values for μ (and q_O2_) were reported [20]. Under an O_2_ concentration of 3.2 kPa (DO < ~1.1 mg/L) *C. necator* grows under O_2_ limitation [33]. From a Monod model of an O_2_-limited chemolithotrophic growth curve, an affinity constant for O_2_ (*K*_O2_) of 0.12 mg/L was determined at the cellular level (at 31 °C, [39]). Under autotrophic conditions and O_2_ limitation, *C. necator* was reported to efficiently accumulate PHB while the formation of protein almost ceased [37]. O_2_ sensitivity of *C. necator* was attributed back to O_2_ inhibition of the involved [NiFe]-hydrogenases. Most [NiFe]-hydrogenases indicates inhibition by O_2_. The β-proteobacterium *C. necator* H16, however, hosts three relatively O_2_-tolerant [NiFe]-hydrogenases involved in energy conversion and H_2_ sensing under aerobic conditions [40].

#### 4.1.2. Technological Limitations in Oxyhydrogen Cultivations

O_2_ transfer from gas into aqueous media is a well-known limitation in cultivation technology due to the low solubility of O_2_. In the cultivation of HOBs, the presence of H_2_ massively complicates processing. O_2_ and H_2_ form explosive gas mixtures with lower and upper explosion limits of 4 and 95.2% H_2_, respectively. The situation is aggravated by the very low ignition energy of 0.016 mJ and is therefore considered to be extremely ignitable. O_2_ concentrations of <6.9 or 5% were previously used as a strategy to maintain the O_2_ concentration below the lower explosion limit (6.9% *v*/*v* in the gas mixture) [10,35]. To promote a sufficiently high DO concentration, extremely high *k*_L_*a*-values [35] or increased pressure were used [10]. Alternatively, electrodes were introduced into the cultivation medium and, by applying sufficient potentials, water was in situ split into O_2_ and H_2_. In non-separated systems, both gaseous substrates were produced in situ and were constantly available [41,42,43,44]. The complete replacement of the terminal acceptor O_2_ by nitrate in autohydrogenotrophic growth of *C. necator* led to low μ of 0.02 h^−1^ [45].

In the present study, an alternative strategy was used: A simplified bioreactor without ignition sources facilitated the safe usage of gas mixtures that were within the explosion range of H_2_ and O_2_. The reactor was driven by an explosion-proof magnetic stirrer, the fume hood was grounded, the lab had an antistatic floor, and scientists were equipped during the gas cultivation with antistatic lab coats and shoes. With the chosen equipment, biomass concentrations of ~13.1 g_CDM_/L were reproducibly obtained despite low *k*_L_*a*-values of 20 to 33 h^−1^. The high biomass concentrations were achieved through tight regulation of the DO. While the DO was kept largely below 1.1 mg/L (Figure 7B,C and Supplementary Information Figure S8), the actual p_i_ of O_2_ was stepwise increased from 0.02 to 0.14 in chemolithotrophic cultivations. Specific growth rates of maximally 0.09 h^−1^ were obtained. The very high μ_max_ of 0.42 h^−1^ (and final biomass concentration of 91 g_CDM_/L) reported by Tanaka et al. [35] is explicable by the high *k*_L_a value of 2970 h^−1^. The study by Tanaka et al. from 1995 is considered the gold standard in autotrophic cultivation. However, complying with modern safety regulations, an oxygen concentration in the gas mixture of <6.9% O_2_ is not considered safe in the published setup for two main reasons: First, back then, a lower explosion limit of 6.9% O_2_ in oxyhydrogen mixtures was stated, whereas currently, a lower explosion limit of 4.8% O_2_ is stated [35,46]. Second, Tanaka et al. used either a bioreactor described as mini-jar fermenter (200 mL) from Able Co., Ltd., Tokyo, Japan [38] or a glass jar fermenter (2 L). From the description and schemes given by them, we assume that both bioreactors had a brush motor on top. Brush motors are considered possible ignition sources, and thus the oxygen concentration in the gas mixture would need to be lower than 4.8% (in the best case several fold lower) to comply with current safety regulations.

#### 4.1.3. Online Analytics in Chemolithotrophic Cultivations

The use of sensor probes in cultivation broths that are constantly gassed by an explosive gas mixture is delicate. Dipping probes certified according to ATEX-RL 2014/34 for ex-zone 0 (Ex II 1G (Ga) IIC T3-T6 required when working with H_2_ and O_2_) are expensive and rare. There are several O_2_ sensors certified for ex-zone 0 but, to the best of our knowledge, none for CO_2_ or H_2_. Here, we used an O_2_-dipping probe based on an optical fiber. As safety measures, the read-out electronics were placed outside of the ventilated hood (safety distance of 2 m). The dipping probe was connected to an equipotential bonding to avoid discharges of static electricity. The dipping probe itself was completely sealed in a steal tube and directly connected to the optical fiber cable. No ignition source was inside the fermenter or the fume hood. The optical dipping probe connected to an equipotential bonding was, to the best of our knowledge, a safe method to measure the DO. High reproducibility of conditions in chemolithotrophic cultivations led to nearly superimposable growth curves illustrated in Figure 7A.

### 4.2. Polyhydroxyalkanoate from CO_2_

Bioplastic from CO_2_ has the dual advantages of CO_2_ assimilation and reduction of plastic products from fossil resources. *C. necator* has been previously proven to accumulate 78 to 82% poly-D-3-hydroxybutyrate (PHB) in two-stage cultivations at cell densities between 60 and 70 g_CDM_/L. The strategy followed a two-stage heterotrophic-chemolithotrophic cultivation using fructose as a carbon source in the heterotrophic phase [7]. The highest production rates of PHB were experienced under oxygen limitation in chemolithotrophic cultures. In heterotrophic cultures, ammonium limitation turned out as the best regime to force PHB accumulation (reviewed in [33]). Similarly, higher PHB contents (up to 82%) under oxygen limitation compared to nitrogen limitation were reported by Mozumder et al. [6]. More recently, *C. necator* was genetically engineered for the biosynthesis of copolymer polyhydroxyalkanoates from CO_2_ without organic precursor molecules [8,9,47]. In the present study (data from cultivation 4), we have observed that after 19 h, the fructose was depleted at a cell dry mass of 0.76g_CDM_/L. The cell dry mass at this timepoint had a PHB content of 9% (equal to 0.068 g_PHB_/L). At the end of cultivation 4, 13.1 g_CDM_/L containing 10.2 g_PHB_/L was obtained. We therefore assume that >98% of the obtained PHB was formed from CO_2_.

### 4.3. Development of the Mineral Medium for Autotrophic Cultivations

TSB is usually used for fast-growing microbes and as standard media for *C. necator*. The complex media contains 20 g/L peptone as organic N- and C-source. The cells would prefer peptone over CO_2_ if both were available in autotrophic cultivations. Therefore, we used TSB for heterotrophic cultivations and mineral media for all autotrophic cultivations and selected heterotrophic cultivations.

The highest biomass concentrations in chemolithotrophic cultivations of *C. necator* were previously reached by Tanaka et al. [35]. However, the use of tap water for preparation masked actual concentrations of trace elements. Repaske and Mayers [12] developed one of the first MMs for chemolithotrophic cultivation of *C. necator* and reported a final biomass concentration of 25 g_CDM_/L. Growth limitations were attributed to depletions in ammonium and trace elements. The media was further adapted in heterotrophic cultivations [48,49] and chemolithotrophic cultivations [50,51]. However, the media used by Lu and Yu [50] led to precipitations upon pH-adjustment. Therefore, we used a medium reported for heterotrophic cultivations of *C. necator* described by Atlić et al. [21] and added tungsten solution (recommended by Cramm [30]) (Table 1). The addition of B12 was motivated by a notion in Pohlmann et al. that *C. necator* H16 is not able to produce vitamin B12. Supplementation with B12 did not improve growth, stressing that the bacteria can bypass vitamin B12-dependent reactions [31].

We decided to add an antibiotic to suppress the growth of contaminating bacteria. Antibiotic resistances of *C. necator* strains are strain-dependent and are of special interest for genetic engineering strategies. The number of antibiotic resistances found in *C. necator* previously complicated the design of plasmids. Wild-type *C. necator* H16 is known to indicate a natural resistance towards the aminoglycoside antibiotic kanamycin applied at concentrations of ≤50 mg/L. Therefore, kanamycin concentrations from 200 to 350 mg/L were previously used for the selection of kanamycin resistance plasmids in *C. necator* H16 [25,51,52,53,54,55]. The presence of tetracycline (tetracycline antibiotic) inhibited the growth of *C. necator* in all used concentrations, also seen for *C. necator* strains isolated from soil [25]. Lacking tetracycline resistance in *C. necator* wild-type strains was exploited for the stabilization of tetracycline-resistance plasmids [53,54].

## 5. Conclusions

CO_2_ assimilation into biodegradable polymers is an attractive strategy to bind and recycle CO_2_, reduce the production of conventional plastic products, and spare fossil resources. The most prominent HOB, *C. necator*, can accumulate polyhydroxybutyrate from CO_2_ to levels of up to 82% of the cell’s dry weight ([6,7] this work). The metabolism of *C. necator* is tractable by genetic engineering, and alternative products (including polyhydroxyalkanoates with altered properties) made from CO_2_ become available [8,10,26,34,56]. One serious bottleneck in the development of new processes based on HOBs is the complexity of oxyhydrogen cultivations with respect to safety aspects, gas mass transfer, and analytics of gaseous substrates dissolved in the aqueous phase. An easy method to test, optimize, and compare growth conditions and strains (engineered and native) was presented. The method built upon an explosion-proof setup for the continuous supply of H_2_, CO_2_, and O_2_. Biomasses of 13.1 g_CD__M_/L with 79% PHB (with approximately 98% of the obtained PHB formed from CO_2_) were easily obtainable with DO as a single controlled parameter. No feeding of salts and no pH and temperature control was required.

## Figures and Tables

**Table 1:** Mineral media (MM) component list.

Substance	g/L	Part
KH_2_PO_4_	1.5	1
Na_2_HPO_4_·2H_2_O	4.5	
(NH_4_)_2_SO_4_	1.5	2
MgSO_4_·7H_2_O	0.2	
NH_4_Fe(III) citrate	0.05	3
CaCl_2_·2H_2_O	0.02	
Tungsten solution	1 mL	4
Fructose	20	5
Trace element solution	1 mL	6
**Tungsten solution**		
Na_2_WO_4_·2H_2_O	0.06	
**Trace element solution**		
H_3_BO_3_	0.6	
CoCl_2_·6H_2_O	0.4	
ZnSO_4_·7H_2_O	0.2	
MnCl_2_·4H_2_O	0.06	
NaMoO_4_·2H_2_O	0.06	
NiCl_2_·6H_2_O	0.4	
CuSO_4_·7H_2_O	0.02	

**Table 2:** Partial pressures of H_2_, CO_2_, O_2_, and total gas flows in individual gas cultivations.

Cultivation	p_H2_:p_CO2_:p_O2_	Total Flow Rate (NmL/min)	Comment
Constant low O_2_ supply			
	Constant 90:8:2	400	-
Constant high O_2_ supply			
	Constant 85:10:5	100	-
Stepwise increase of O_2_ guided by the biomass			
	start 90:8:2 end 80:8:12	400	Intermittent gas compositions are in the Supplementary Data file.
Stepwise increase of O_2_ guided by the DO probe			
Cultivation 1	start 85:10:2 end 71:7:21	start 97 end 140	All intermittent gas compositions are in the Supplementary Data file.
Cultivation 2	start 85:10:2 end 80:8:12	start 97 end 250	
Cultivation 3	start 85:10:2 end 81:8:11	start 97 end 250	
Cultivation 4	start 85:10:2 end 81:8:11	start 97 end 250	

**Table 3:** Comparison of different O_2_ supply strategies. Effect of starting p_O2_ and p_O2_ supply method.

Cultivation	p_O2_ (atm)	μ_max_ (h^−1^)	Final OD_600_	Comment
Constant low O_2_ supply	constant at 0.02	0.008	5.0	Figure 6
Constant high O_2_ supply	constant at 0.05	not determined	1.3	Figure S7
Stepwise increase of O_2_ guided by the biomass	start 0.02 end 0.12	0.055	26.5	Figure 6; biomass conc. measured offline, O_2_ supply increased manually
Stepwise increase of O_2_ guided by the DO probe (cultivations 3, 4) *	start 0.02 ± 0.00 highest 0.19 ± 0.02 end 0.11 ± 0.00	0.095 ± 0.01	53.5 ± 1.5	Figure 7; DO measured online, O_2_ supply increased manually

* Note that cultivations 1 and 2 stopped at earlier timepoints and were hence not used for the summary. O_2_ supply is critical from a biological and technological point of view.

## Data Availability

The data presented in this study is available in [Supplementary data.xls] file.

## Supplementary Materials

The following supporting information can be downloaded at: https://www.mdpi.com/article/10.3390/bioengineering9050204/s1, Figure S1: Wavelength scan of water, TSB and mineral media; Figure S2: Correlation of OD_600_ to CDM in *C. necator* cultures; Figure S3: Gassing and bubble formation in the stirred 1000 mL DURAN® GLS 80 bioreactor; Figure S4: *k*_L_*a* values for the 1 L DURAN® GLS 80 bioreactor at gas flows of 100, 200 and 400 mL at 340 rpm; Figure S5: Heterotrophic cultivations at room temperature (22–24 °C, orange dots) and at 30 °C (grey dots) in 1 L baffled flasks (magnetic bar, stirring at 400 rpm); Figure S6: Determination of maximal growth rates from chemoautotrophic cultivations depicted in Figure 6 and Figure 7 (main part); Figure S7: Time curve of a gas fermentation with constant high O_2_ supply. OD_600_ values (black circles) and p_O2_ (blue line); Figure S8: Time curves of gas cultivations 1 (panel A) and 2 (panel B) with manual DO control using a dipping probe (supplementing Figure 7A main part); Table S1: Maximal growth rates (μmax h^−1^) obtained at room temperature or 30 °C.
