# Supplementary material for: Lab-Scale Cultivation of Cupriavidus necator on Explosive Gas Mixtures: Carbon Dioxide Fixation into Polyhydroxybutyrate
Source: Bioengineering (Basel). 2022 May 10;9(5):204. doi: 10.3390/bioengineering9050204 (PMC9138072; doi:10.3390/bioengineering9050204)
Supplement: Supplementary file 1 [file bioengineering-09-00204-s001.zip › Supplementary Information_revision.pdf]

Supplementary Information

Lab-Scale Cultivation of *Cupriavidus necator* on Explosive Gas Mixtures: Carbon Dioxide Fixation into Polyhydroxyalkanoate

Vera Lambauer[a,b], Regina Kratzer[b,a]

[a]Austrian Centre of Industrial Biotechnology (ACIB), 8010 Graz, Austria.
[b]Institute of Biotechnology and Biochemical Engineering, Graz University of Technology, NAWI Graz, 8010 Graz, Austria.

*Corresponding author phone +43 316 873 8412, e-mail: regina.kratzer@tugraz.at

Content

1. Optical density (OD_{600}) cell-dry mass (CDM) correlation

Figure S1. Wavelength scan of water, TSB and mineral media.
Figure S2. Correlation of OD_{600} to CDM in *C. necator* cultures.

2. Determination of the volumetric oxygen transfer coefficient (k_La)

Figure S3. Gassing and bubble formation in the stirred 1L DURAN® GLS 80 bioreactor. A magnetic anchor stirrer (reactor cap GLS 80) and an PTFE frit (Agilent) were used for stirring and gassing.
Figure S4. k_La values for the 1L DURAN® GLS 80 bioreactor at gas flows of 100, 200 and 400mL at 340rpm.

3. Effect of temperature on growth

Table S1. Maximal growth rates (μ_{max} h^{-1}) obtained at room temperature or 30°C (mineral media, cultivation time 3 days).
Figure S5. Comparison of heterotrophic cultivations at room temperature (22 – 24°C) and at 30°C.

4. Chemoautotrophic cultivation (oxyhydrogen fermentation)

Figure S6. Determination of maximal growth rates from chemoautotrophic cultivations depicted in Figures 6, 7 (main part). Crosses show growth rates from cultivations with manual DO control guided by the O_2 sensor (light blue crosses constant and low H_2 and CO_2, orange and yellow crosses increase in H_2 and CO_2 after ~60h). Blue diamonds from a cultivation with manual DO control guided by the biomass formation, grey triangles from a cultivation with constant gas composition.
Figure S7. Time curve of a gas fermentation with constant high O_2 supply. OD_{600} values (black circles) and pO_2 (blue line).
Figure S8. Time curves of gas cultivations 1 (panel A) and 2 (panel B) with manual DO control using a dipping probe (supplementing Figure 7A main part). Panel B shows gas cultivation 2 with H_2 and CO_2 flows increased to 200 and 20NmL/min at 57h. (OD_{600} values circles, p_{O2} blue line, DO in % of saturation, blue dots, $(NH_4)_2SO_4$ white triangles).

1. Optical density (OD_{600}) cell-dry mass (CDM) correlation

The wavelength for optical cell density measurements was set to 600nm according to wave length scans of pure media (Figure S1). The scan was performed on a Beckman DU-800 spectrophotometer. Cell dry mass (CDM) was determined gravimetrically. 5mL of the cell suspension were filled in a previously dried vial. 5mL of the media were transferred into a further vial and used as reference. All vials were dried at 105°C over night. After cooling down in a dessicator for at least 4-6 h, the cell dry mass was determined by substracting the mass of the cells with media from the mass of the media alone. OD_{600} and CDM data were correlated (Figure S2) to facilitate fast CDM estimation during fermentations.

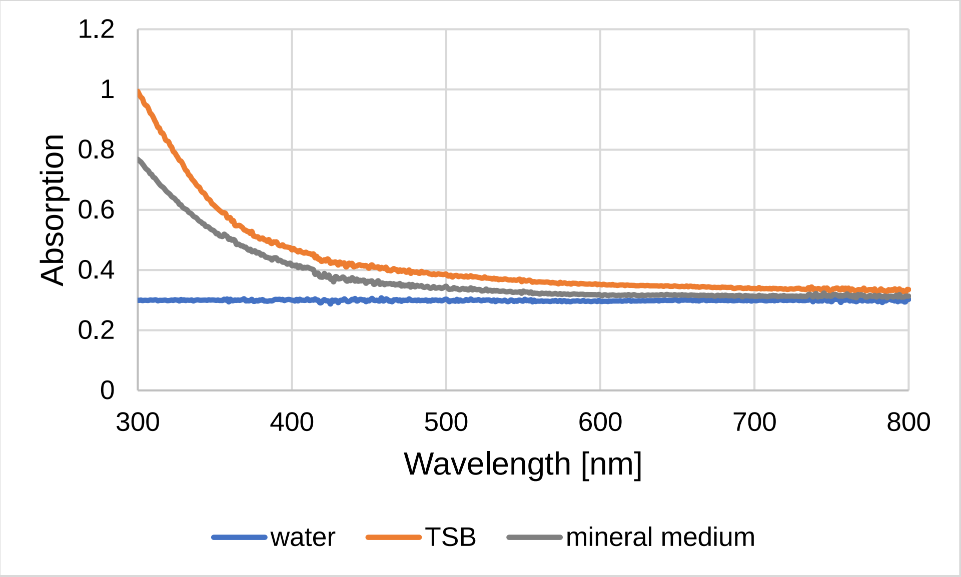

<u>Figure S1</u>. Wavelength scan of water, TSB and mineral media. The scan was performed on a Beckman DU-800 spectrophotometer.

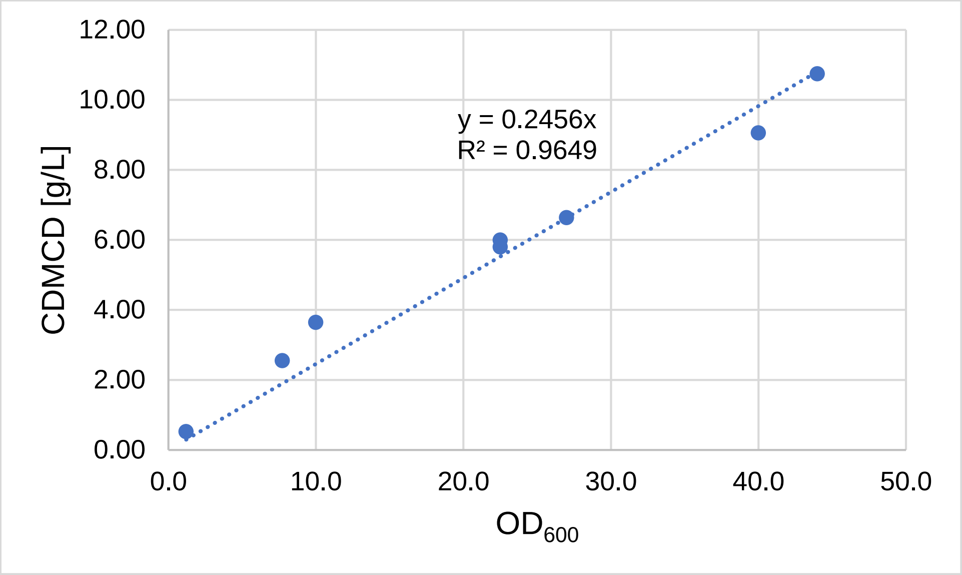

Figure S2. Correlation of OD_{600} to CDM in *C. necator* cultures.

2. Determination of the volumetric oxygen transfer coefficient (k_La)

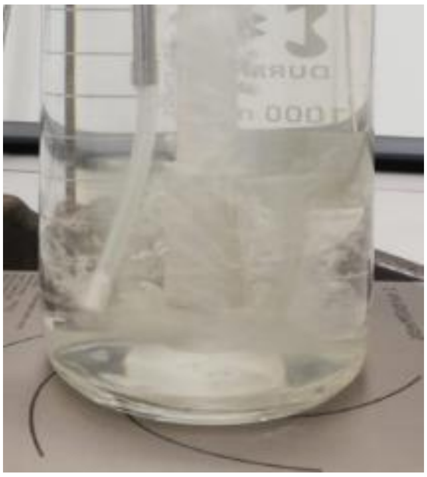

Figure S3. Gassing and bubble formation in the stirred 1000 mL DURAN® GLS 80 bioreactor. A magnetic anchor stirrer (reactor cap GLS 80) and an PTFE frit (Agilent) were used for stirring and gassing.

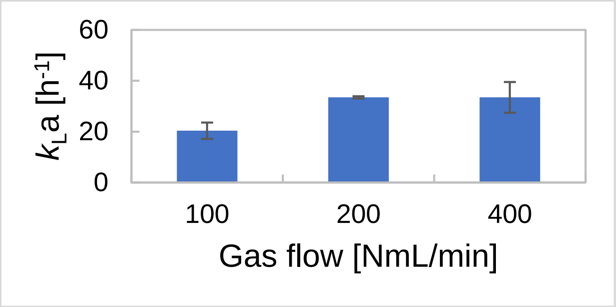

Figure S4. k_La values for the 1L DURAN® GLS 80 bioreactor at gas flows of 100, 200 and 400mL at 340rpm.

3. Optimization of growth conditions

3.1 Effect of temperature on growth

Table S1. Maximal growth rates (μ_{max} h^{-1}) obtained at room temperature or 30°C (mineral media, cultivation time 3 days).

Cultivation flask (stirring, shaking)	Temperature	μ_{max} h^{-1}*
1L baffled flask (magnetic bar, 400rpm)	30°C (thermostatized)	0.179 ± 0.014
1L baffled flask (magnetic bar, 400rpm)	22-24°C (room temperature)	0.132 ± 0.006
300mL baffled flask (orbitally shaken, 110rpm)	30°C (thermostatized)	0.172 ± 0.007
1 L DURAN® GLS 80 bottle (magnetic anchor stirring, 400 rpm)	22-24°C (room temperature)	0.120 ± 0.015

*Mean value and deviation from the mean of two independent cultivations.

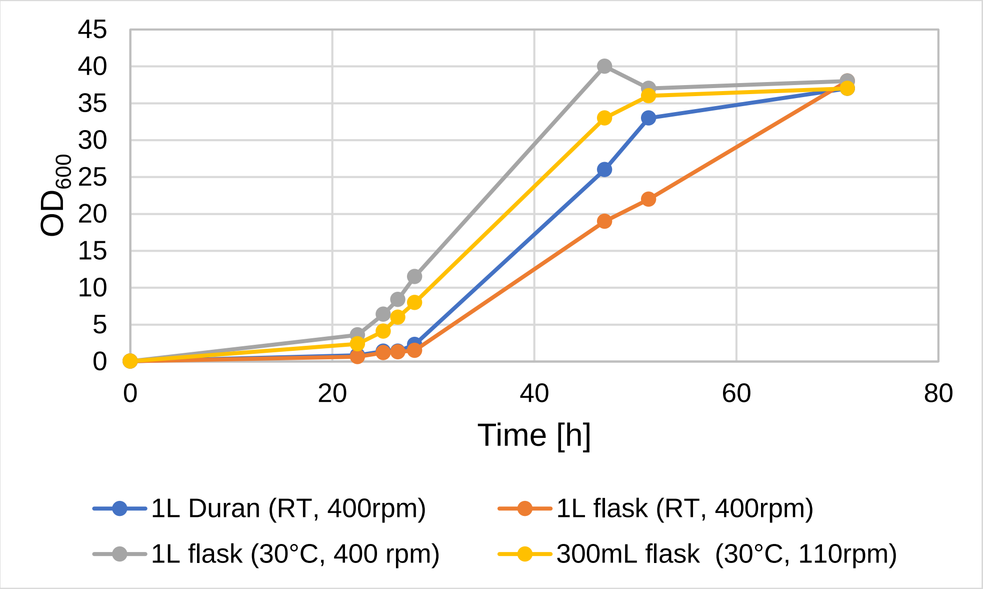

Figure S5. Heterotrophic cultivations at room temperature (22 – 24°C, orange dots) and at 30°C (grey dots) in 1L baffled flasks (magnetic bar, stirring at 400rpm). Compared to cultivation at room temperature in the 1L DURAN® GLS 80 bottle (400 rpm) (blue dots) and cultivation at 30°C in 300mL shaken flask (orbitally shaken at 110rpm) (yellow dots).

4. Chemoautotrophic cultivation (oxyhydrogen fermentation)

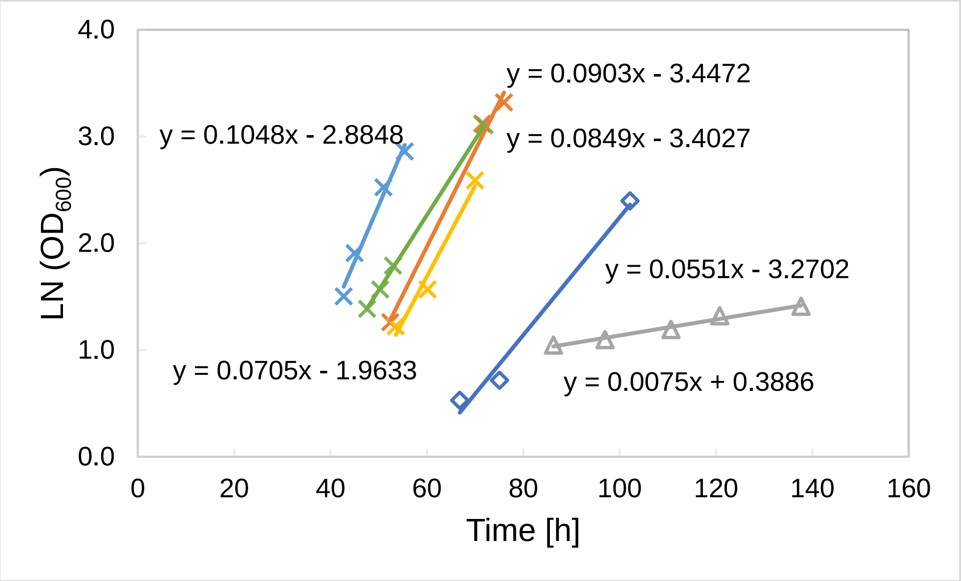

Figure S6. Determination of maximal growth rates from chemoautotrophic cultivations depicted in Figures 6, 7 (main part). Crosses show growth rates from cultivations with manual DO control guided by the O_2 sensor (light blue crosses gas cultivation 4, green crosses gas cultivation 1, orange crosses gas cultivation gas cultivation 2, yellow crosses gas cultivation 3; gas cultivations 2 and 3 increase in H_2 and CO_2 after ~60 h, gas cultivation 4 increase in H_2 and CO_2 after 45 h). Blue diamonds from a cultivation with manual DO control guided by the biomass formation, grey triangles from a cultivation with constant gas composition.

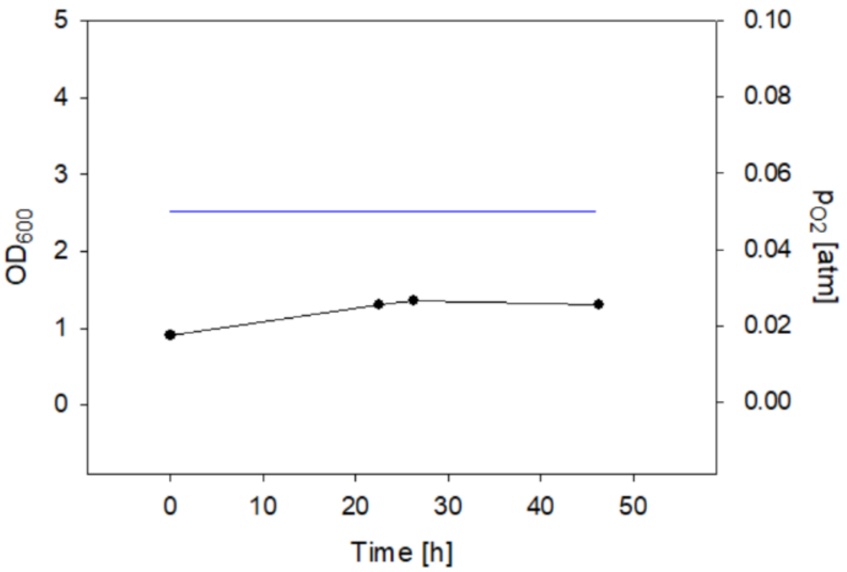

Figure S7. Time curve of a gas fermentation with constant high O_2 supply. OD_{600} values (black circles) and pO_2 (blue line).

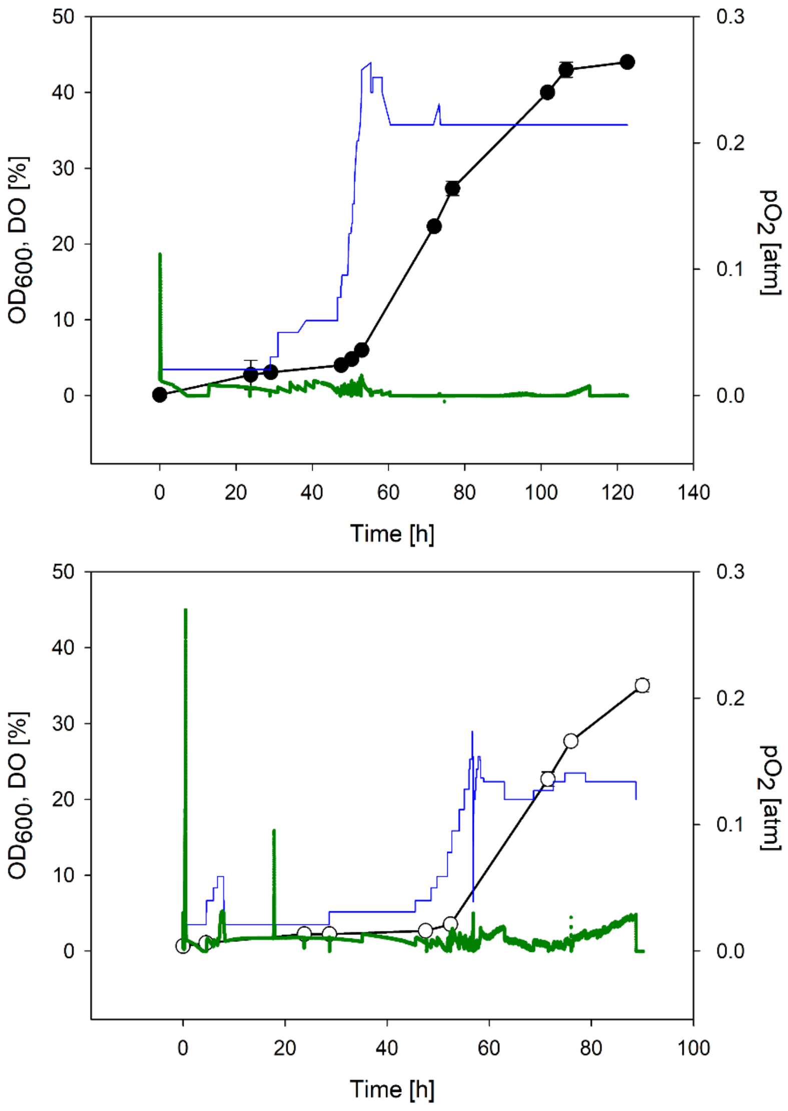

Figure S8. Time curves of gas cultivations 1 (panel A) and 2 (panel B) with manual DO control using a dipping probe (supplementing Figure 7A main part). Panel B shows gas cultivation 2 with H_2 and CO_2 flows increased to 200 and 20NmL/min at 57h. (OD_{600} values circles, p_{O2} blue line, DO in % of saturation blue dots seen as thick line.)
